# Utilization of DES-Lignin as a Bio-Based Hydrophilicity Promoter in the Fabrication of Antioxidant Polyethersulfone Membranes

**DOI:** 10.3390/membranes8030080

**Published:** 2018-09-08

**Authors:** Mohammadamin Esmaeili, Ikenna Anugwom, Mika Mänttäri, Mari Kallioinen

**Affiliations:** 1 LUT School of Engineering Science, Department of Separation and Purification Technology, Lappeenranta University of Technology, P.O. Box 20, Lappeenranta FIN-53851, Finland; mika.manttari@lut.fi; 2 LUT Re-Source Platform, Lappeenranta University of Technology, P.O. Box 20, 53851 Lappeenranta, Finland; ikenna.anugwom@lut.fi (I.A.); mari.kallioinen@lut.fi (M.K.)

**Keywords:** lignin, deep eutectic solvent (DES), antioxidant, hydrophilic adhesion promoter, green solvent

## Abstract

Enhancement of membrane permeability at no detriment of its other performances, e.g., selectivity, is a goal-directed objective in membrane fabrication. A novel antioxidant DES-lignin (lignin extracted from birch wood by using a deep eutectic solvent) polyethersulfone (PES) membrane, containing 0–1 wt % DES-lignin, was fabricated with the phase inversion technique. The performance and morphology of the fabricated membranes were characterized by a pure water flux, polyethylene glycol (PEG) retention, Fourier transform infrared spectroscopy, scanning electron microscopy, and contact angle measurements. Membranes with less negative charge and better hydrophilicity were obtained when the DES-lignin content in the polymer solution was increased. With the highest dosage, the incorporation of DES-lignin in the membrane matrix improved the membrane permeability by 29.4% compared to a pure PES membrane. Moreover, no leakage of DES-lignin from the membrane structure was observed, indicating good compatibility of DES-lignin with the PES structure. It was also found that the improvement of both rejection and pure water flux could be achieved by using a small dosage of DES-lignin (0.25 wt %) in membrane fabrication. The membranes incorporated with DES-lignin showed higher DPPH (2,2-diphenyl-1-picrylhydrazyl) and ABTS (2,2-Azino-bis (3-ethylbenzothiazoline-6-sulfonic acid)) scavenging activity compared to the pure membrane, where 2.6 and 1.1 times higher DPPH and ABTS scavenging activity was observed with the highest DES-lignin content (1 wt %). Thus, the results of this study demonstrate well the feasibility of utilizing DES-lignin as an antioxidant bio-based hydrophilicity promoter in the fabrication of ultrafiltration membranes.

## 1. Introduction

To fulfill the different aspects of green chemistry and to achieve full use of lignin from its primitive resources, the necessity of developing green methods for the fractionation of lignocellulosic materials is obvious. Ionic liquids (ILs) are comprised of large organic cations and inorganic or organic anions that exist as liquids at relatively low temperatures (lower than 100 ${}^{{}^{\circ}}$C) [1,2]. Compared to traditional organic solvents, ILs have attractive properties, such as thermal stability, versatility, self-organized structure, high solvent capacity, insignificant vapor pressure (reduction in volatile organic compound emissions), thermal and chemical stability, recyclability and often non-flammability, which make them suitable to be considered as green solvents in lignocellulosic material dissolution [3,4,5]. However, ILs have also several weaknesses, such as high cost, low moisture tolerance, biodegradability that is not very notable, and the inclusion of synthesis mostly from oil-based chemicals [5,6,7]. Moreover, the possible toxicity of ILs is a factor of particular importance that should be taken into account in the case of practical applications in a large scale [7,8]. These downsides, together with the requirement of a large amount of salts and solvents in their synthesis in order to exchange the anions, entirely hinder their industrial inception [9]. Deep eutectic solvents (DES) as a new generation of ILs have emerged as a renewable and biodegradable green solvent with almost all the advantages of ILs in the dissociation of lignocellulosic material, as well as their considerably lower toxicity and price [9]. In general, the interactions between their hydrogen bonds of an HBD (hydrogen bond donor) and an HBA (hydrogen bond acceptor) mixture results in the formation of DESs. Consequently, the melting temperature of the formed eutectic mixture is lower compared to that of its constituents [10].

Deep eutectic solvents can play a substantial role in pulp and paper and recycling industries. The treatment of wood chips or annual plants with DESs in delignification or pulping processes can remarkably reduce the required energy for pulp production. Furthermore, there is a potential to utilize deep eutectic solvents in hydrolysis or dissolution of certain components of lignocellulosic biomass, such as lignin, under mild conditions, which in turn can minimize the risk of further degradation of these components [11]. In addition, higher solubility of lignin in deep eutectic solvents (DES delignification) compared to the other delignification processes (e.g., oxygen delignification) has been reported in different studies [12,13].

Polyethersulfone (PES) is considered as one of the most favorable polymeric materials in the fabrication of ultrafiltration membrane, due to its outstanding chemical and thermal stability, appropriate mechanical strength, and film-forming properties [14,15,16]. Furthermore, it has been implemented as the main base membrane material in various substantial separation processes, such as concentration, extraction, purification, and hemodialysis [15]. Even with its attractive properties, a PES membrane is susceptible to fouling due to its relatively low hydrophilic nature, e.g., in the treatment of biorefinery streams and pulp and paper industry process waters [17,18]. Thus, the incorporation of different organic or inorganic additives, such as polyvinyl pyrrolidone (PVP), polyvinyl alcohol (PVA), and polyethylene glycol (PEG), in the dope solution before phase inversion can be considered a promising approach to eliminate this downside, which in turn can enhance the membrane performance [16].

The existing literature on the application of lignin and its derivatives in different fields of science is extensive and focuses particularly on the utilization of this natural adhesion promoter in water treatment for removing different pollutants [19] or as an additive, such as binder, dispersant, and emulsifier [10]. There is a relatively small body of literature that is concerned with the fabrication or modification of membranes by native lignin. Ameliorating the performance of a membrane by using natural additives, such as lignin, which offers renewability, availability, and low cost advantages, is attentive due to environmental aspects in terms of biodegradability and of the reduction of membrane fabrication costs compared to synthetic additives [20]. In addition, it has been highlighted that polymeric hydrophilic additives like PEG might leach out from the membrane structure during membrane formation and filtration processes due to their affinity to water [20,21,22]. Leaching from the membrane structure can have detrimental health effects especially in the food separation industry, as it may interact with food materials. Moreover, such additives are not the ideal ones in the modification of thin-film composite membranes, as a remarkable part of the weight of the thin film can be removed, which in turn reduces the half-life of the membrane [20].

Cleaning operation is an inevitable part of membrane processes, playing a vital role in the aging of the membrane. Quick back flushing with a cleaning chemicals, such as chlorine and hypochlorite, is one of the most commonly used methods in membrane cleaning processes, which can affect the degradation of the membrane material by radical oxidation of polymer chains and thus shorten its life time and increase the operation costs [23]. Preparation and modification of the membrane with antioxidant characteristics can be an appropriate way to reduce the aging of the membrane caused by regular cleaning or in gas separation processes that are in contact with oxygen [24]. Furthermore, having an antioxidant membrane can reduce the possible changes that might happen during storage in different conditions. Above all, there is a great amount of published studies describing the antibacterial role of phenolic antioxidants [25,26], and they can be used preferably as additives in membrane fabrication to reduce biofouling due to the formation of a biofilm on the surface of the membranes.

So far, previous studies have demonstrated the potential of lignin as a natural plant-based polymer in improving the toughness of polymer composites [27,28]. Lignin derivatives and alkaline lignin have been used as additives in membrane separation and fabrication. For example, Knyazkova and Zhurayev confirmed the effectiveness of sulphate lignin in the reduction of flux decline when separation of low molecular mass organic solutes was conducted by cellulose acetate membranes [29]. Lignosulfonates have been incorporated in the fabrication of polysulfone membrane to impart electrolyte transfer. This modification reduced the formation of macrovoids and facilitated the formation of larger surface pores [30]. In another work, Navarez et al. modified a cellulose triacetate membrane with three diffident propionated lignins (Kraft, Organosolv and Hydrolytic), and the membrane performance was examined by groundwater containing a high concentration of different ions. In general, they report that both wettability and flux were reduced as the propionation of lignin increased in the fabricated nanocomposites [31]. Alkali lignin (28 kDa; 0.125 and 0.5 wt %) has also been used as an additive in the synthesis of an ultrafiltration polysulfone membrane [20], and the membrane morphology and performance, as well as the tensile strength, porosity, glass transition temperature, thermal properties, and other mechanical properties of the fabricated membrane [16,32], were studied and compared with PEG (10 kDa) and PVP (29 kDa) as the most two common additives used in membrane modification. It was found that alkali lignin improved the thermal stability and increased the bulk porosity and permeability of the prepared membranes. However, the alkali lignin-modified membranes revealed a high molecular cut-off value as the best separation factor (actual rejection of ∼ 93%) was obtained when PVP-360 kDa was utilized as a model compound [20]. Moreover, in another studies, alkali lignin (28 kDa) [33] and sodium lignin sulfonate [34] were utilized as additives in a polysulfone substrate layer of reverse osmosis membrane for forward osmosis (FO) applications. In the most recent study by Zhang et al., the water permeation, salt rejection, and fouling resistance of reverse osmosis (RO) were improved by depositing commercial alkaline lignin on the surface of a commercial RO membrane [35].

In this study, DES-lignin as a hydrophilic adhesion promoter was initially extracted from birch wood by using a deep eutectic solvent (DES) and it was then used as an additive to fabricate a novel antioxidant ultrafiltration polyethersulfone membrane. The performance of the prepared membranes was evaluated on the basis of pure water flux and PEG rejection. In addition, the effect of different dosages of DES-lignin, the antioxidant activity, hydrophilicity, surface charge, FTIR, and the degree of ISO brightness of the fabricated membranes were appraised. The study aims at contributing to this growing area of research by utilizing natural polymeric materials from lignocellulosic sources as additives in the fabrication and modification of membranes with better performance and antioxidant characteristics.

## 2. Materials and Methods

### 2.1. Chemicals

Polyethylene glycol (PEG, approx. Mw. 35,000 g/mol, CAS: 25322-68-3, Sigma-Aldrich, Saint Louis, MO, USA) was purchased from Fluka AG (Buchs, Switzerland) and used as the model compound for the rejection study. The following chemicals were used in the preparation of the membrane casting solution: Polyethersulfone (58,000 g/mol, Goodfellow Cambridge Ltd., Huntingdon, UK), *N*-Methyl-2-Pyrrolidone (NMP, CAS: 872-50-4) as the basic polymer and solvent provided by Merck Co. (Darmstadt, Germany), and *N*,*N’*-dimethylformamide (DMF) (Fluka Chemie AG, Buchs, Switzerland, ≥99%). 2,2-Azino-bis (3-ethylbenzothiazoline-6-sulfonic acid) diammonium salt (ABTS) (Saint Louis, MO, USA, CAS: 30931-67-0) and 2,2-diphenyl-1-picryl hydrazyl (DPPH) (Saint Louis, MO, USA, CAS: 1896-66-4) were purchased from Sigma-Aldrich, and used as received. Dimethyl sulfoxide (DMSO, CAS: 6768-5) was obtained from AppliChem GmbH (Darmstadt, Germany). Ultra-pure deionized water (DI, 15 MΩ) was obtained from a CENTRA-R 60/120 system (Elga purification system, Veolia Water, Bucks, UK), and used in all experiments and membrane preparation stages.

### 2.2. General Procedure for DES Preparation

The preparation of DES was implemented by the combination of choline chloride (VWR, >98% purity, CAS number 67-48-1) and lactic acid (VWR, CAS: 50-21-5) at the molar ratio of 1:9, followed by rigorous agitation for 2 h at 85 ${}^{{}^{\circ}}$C. Once a homogeneous and transparent liquid without a presence of solid particles was achieved, the resulting clear liquid was laid aside in a desiccator at room temperature to be cooled gradually without any moisture absorption.

### 2.3. Lignin Extraction

The extraction of lignin from wood was conducted according to the following extraction procedure. Briefly, 20 g of wood chips with specific sizes of (5 × 5 × 0.2 mm) was added to 200 g of a prepared DES solution in a 500 mL serum bottle and heated immediately after that to 100 ${}^{{}^{\circ}}$C in an oil bath. The dispersion was kept heated at this temperature with stirring for 18 h. Subsequently, the cooked mixture was filtrated through a filter paper in a ceramic Büchner funnel under a vacuum and washed thoroughly with a mixed solution of acetone and water (volume ratio 1:9). The filtrate was collected and the solvents, except for DES, were evaporated by using a rotary evaporator. Afterwards, deionized water was added to the remaining solution containing lignin and DES in order to precipitate the lignin. Finally, the lignin was separated by a centrifuge and subsequently freeze-dried. The purity of lignin based on the Klason lignin analyses was 80% and the rest of the dry matter consists of mainly polymeric carbohydrates.

### 2.4. Membrane Preparation

Asymmetric PES-based and lignin-modified membranes were fabricated with the wet phase inversion technique according to the procedure described in detail by Mulder [36]. The preparation o7f the dope solution was initiated by dissolving a desired amount of extracted lignin (0, 0.25, 0.5, and 1 wt % based on the weight ratio of lignin to the PES solution) into a mixture of *N*-Methyl-2-Pyrrolidone (NMP) and DMF ( ratio 3:1 NMP:DMF) as solvents under constant agitation at room temperature for 6 h. Once the dissolution of additives was obtained completely, an adequate amount of dry PES beads was added gradually to the mixture to attain the desired polymer concentration (20 wt %). After being fully mixed, the homogeneous dope solution was laid aside to degas overnight in order to eliminate any air bubbles, which might be present in the dope solution. The polymer solution was then cast on a glass plate (characterization tests) and on polyester (performance test) by using a film applicator with a thickness of 200 μm. After that, the glass plate was immersed immediately in a coagulation bath consisting of DI at 10 ${}^{{}^{\circ}}$C in order to initiate coagulation. After 30 min, the formed membrane was taken out and put into a new coagulation bath at the same temperature and kept overnight to remove the remaining solvent inside the membrane completely. In addition, the polymer solution was kept in a dark environment to avoid an aging process [37].

### 2.5. Membrane Characterization

#### 2.5.1. Molar Mass Distribution Analysis

The molecular weight distribution of DES-lignin was analyzed by gel permeation chromatography on the HPLC system (Shimadzu HPLC, Kyoto, Japan), which comprises the following parts: a system controller SCL-10AVP, an on-line degasser DGU-14A, a low-pressure gradient valve FCV-10ALVP, an HPLC pump LC-10ATVP, an autosampler SIL-20AHT, and a column oven CTO-10ACVP. The system was equipped with a sequentially connected guard column (50 mm × 7.8 mm) and two Jordi Gel DVB 500A (300 mm × 7.8 mm) columns in a series. Separations were run by using THF with 1% acetic acid as an eluent with a constant flow rate of 0.8 mL min${}^{-1}$. The detector parameters were as follows: an HPLC nebulizer; 40 ${}^{{}^{\circ}}$C; an air pressure of 3.5 bar; a gain of 3; no-split mode. The injection volume of the autosampler was 50 μL. The oven column was set to 40 ${}^{{}^{\circ}}$C. Polystyrene Standards (Perkin-Elmer, Norwalk, CT, USA) were used to calibrate the columns. Chromatogram evaluation was implemented by commercial software for GPC analysis CLASS-VP Version 1.03 (Shimadzu).

#### 2.5.2. Color Parameter Measurements

The color parameters (L${}^{*}$, a${}^{*}$ and b${}^{*}$) and ISO brightness (R457 C) of virgin and lignin-modified membranes were measured with a color meter by using Lorenzen & Wettre Elrepho Spectrophotometer 2000. The calibration was implemented by a white tile prior to measurement. The color parameters of each piece of membrane samples were the average of four randomly selected spots. Two membrane coupons were used for each type of fabricated membrane. The light source used in this study was D65 and C light.

#### 2.5.3. Determination of Antioxidant Activity

The antioxidant activity of fabricated membranes was evaluated by using DPPH (2,2-diphenyl-1-picrylhydrazyl) and ABTS free radical scavenging assay described by Sun et al. [38], with some modification. A portion of 30 mg membrane samples were mixed with 5 mL of DMSO in glass tubes and sonicated in a bath type sonicator for 3 h, and subsequently centrifuged (SkySpin CM-6MT centrifuge, ELMI Ltd., Riga, Latvia) at 3500 rpm for 10 min. Afterwards, 7.4 mg of DPPH was dissolved in 100 mL of methanol for an absorbance of 1.8 at 520 nm. After that, the absorption intensity of 4 mL of DPPH and an aliquot of 800 μL of supernatant was measured at 520 nm. Equation (1) was used to determine the percentage of DPPH free radical quenching activity (*S*(%)):

(1) $$S\left(\%\right)=\frac{A_{control}-A_{sample}}{A_{control}}\times 100$$

where *A*${}_{control}$ and *A*${}_{sample}$ are the absorbance of methanoic solution of DPPH and the mixure of sample extract and DPPH solution at 520 nm, respectively.

For analyzing the ABTS free radical scavenging activity, a mixture of 10 mL of 7 mM ABTS aqueous solution in 10 mL of 2.54 mM potassium persulfate aqueous solution was first prepared and kept in the dark for 16 h (ABTS${}^{*}$). After that, the absorbance of ABTS${}^{*}$ was adjusted to 0.7 ± 0.02 by diluting with DMSO. Finally, the absorbance of an aliquot of 40 μL supernatant and 4 mL diluted ABTS${}^{*}$ after 5 min vortex was measured at 770 nm with a UV-vis spectrometer (Jasco V-670 Spectrophotometer, Tokyo, Japan). DMSO was used as the blank and the ABTS${}^{*}$ antioxidant capacity was evaluated based on Equation (2):

(2) $$ABTS^{*}antioxidantcapacity\left(\%\right)=\frac{A_{control}-A_{sample}}{A_{control}}\times 100$$

where *A*${}_{control}$ and *A*${}_{sample}$ are the absorbance of the mixture of ABTS${}^{*}$ with DMSO and the mixture of sample extract and ABTS${}^{*}$ solution at 770 nm, respectively.

#### 2.5.4. Fourier Transform Infrared Spectroscopy

To characterize the presence of lignin in modified membranes, FTIR spectra of the virgin PES membrane and the ones modified with lignin were measured by using a Perkin Elmer Frontier spectrometer with a universal ATR module (Diamond crystal). FTIR spectra of six random spots from each membrane sample were measured in the 4000–400 cm${}^{-1}$ wavenumber range with the resolution of 4 cm${}^{-1}$. All spectra were the acquisition of 4 scans with the data interval of 1 cm${}^{-1}$ at the absorbance mode. At the final step, the co-added spectra were processed with ATR correction and baseline correction, and finally normalized.

#### 2.5.5. Scanning Electron Microscopy

The cross-sectional morphology of the membranes was studied qualitatively with a scanning electron microscope (Hitachi SU 3500, Tokyo, Japan) at the acceleration voltage of 15 kV in a high vacuum condition. Narrow strips of membranes were prepared and soaked in DI water overnight and then snapped under liquid nitrogen with the aid of two pairs of forceps to obtain a clean cut. Prior to the sputtering process, the samples were dried in air to eliminate excess ice and water [39]. The membrane samples were subsequently coated with a thin layer of gold by using the Edwards Scancoat six Pirani 501 sputter coating system (Edwards High Vacuum International, Crawley, UK). Finally, the cleaved edge was examined perpendicular to the cut plane by SEM.

#### 2.5.6. Casting Solution Viscosity Measurement

Regarding the considerable role of casting solution viscosity on pore formation and the final structure of the membrane, the dynamic viscosities of the dope solutions were measured with a Modular Compact Rheometer MCR 302 (Anton-Paar, Graz, Austria, PP50/P2 spindle) at 20 ${}^{{}^{\circ}}$C.

#### 2.5.7. Contact Angle Measurements

The static contact angles of the fabricated membranes were measured by sessile drop methods. Roughly 5 μL of DI water was placed on the surface of the membrane samples with the aid of a micro syringe at room temperature. To increase the reliability of the measurements, the contact angles of at least 10 independent points for each sample were tested, and the average of recorded data was considered as the final CA. The CA was measured by using a KSV CAM 101 instrument (KSV Instruments Ltd., Helsinki, Finland) connected to a CCD camera (DMK 21F04, The Imaging Source Europe GmbH, Bremen, Germany). The captured images were treated by curve fitting analysis with CAM 2008 software in order to determine the CA.

#### 2.5.8. Surface Charge Measurements

The membrane surface charge is one of the most relevant influencing factors in aqueous filtration processes, and it is related to the zeta potential. The zeta potential of the virgin membrane and the modified ones with lignin was measured by an electro-kinetic analyzer (SurPASS, Anton Paar, Graz, Austria) with an adjustable gap cell and using 1 mM KCl as the background electrolyte solution. The membrane pieces were stored in DI water in a fridge (about 4 ${}^{{}^{\circ}}$C) prior to use. The solution pH was first shifted to about 8 by a dilute KOH solution and then automatically titrated from 8 to 3 by using a 0.05 M HCl solution during the analysis. Finally, the zeta potential was calculated from streaming current measurement according to the classic Helmholtz–Smoluchowski equation [40].

(3) $$\zeta =\frac{E_{S}}{\Delta P}\frac{\eta \kappa }{\varepsilon_{r}\varepsilon_{0}}$$

where *E${}_{S}$* is the tangential streaming potential, *ΔP* the pressure gradient, κ the conductance of the electrolyte solution, η liquid viscosity, ε${}_{0}$ vacuum permittivity, and ε${}_{r}$ is the relative dielectric constant.

### 2.6. Experimental Design and Procedure

#### Pure Water Flux and Performance Analysis

The pure water flux and PEG solution rejection for both virgin and lignin-modified membranes were measured in a batch mode with a dead-end filtration system by using an Amicon solvent-resistance stirred cell (Millipore, Burlington, MA, USA; Cat No.: XFUF07611, diameter of mixer: 60 mm) with the internal diameter of 76 mm. The effective filtration area available for filtration was 38 cm${}^{2}$. First, the pressure was gradually increased to 5 bar, and the membrane samples were then compacted at this temperature for 20 min. Afterwards, the pure water flux was recorded at 1, 2, and 3 bar for 15 min at 25 ± 1 ${}^{{}^{\circ}}$C, and the pure water flux ($J_{w}$ (L m${}^{2}$ h${}^{-1}$)) over the measured time intervals was calculated by using Equation (4):

(4) $$J_{w}=\left(\frac{Q}{A\times t}\right)$$

where *A*, *t*, and *Q* are the effective area of filtration (m${}^{2}$), sampling time (h), and volumetric flow rate of the permeate (L), respectively. After that, the Amicon cell was rinsed with 100 g of PEG solution in order to eliminate the effect of remaining water in the dilution of the PEG solution. Afterwards, the rejection of the PEG solution (300 ppm) after 5 min through the membrane samples at 25 ± 1 ${}^{{}^{\circ}}$C was calculated by using Equation (5):

(5) $$R\left(\%\right)=\left(1-\frac{2\times c_{p}}{c_{f}+c_{r}}\right)\times 100$$

where *C${}_{p}$*, *C${}_{f}$*, and *C${}_{r}$* are the concentration of PEG in the permeate, the initial feed (i.e., *t* = 0), and the retentate (at the end of filtration process), respectively.

## 3. Results and Discussion

### 3.1. Characterization of DES-Lignin

**Figure 1:**
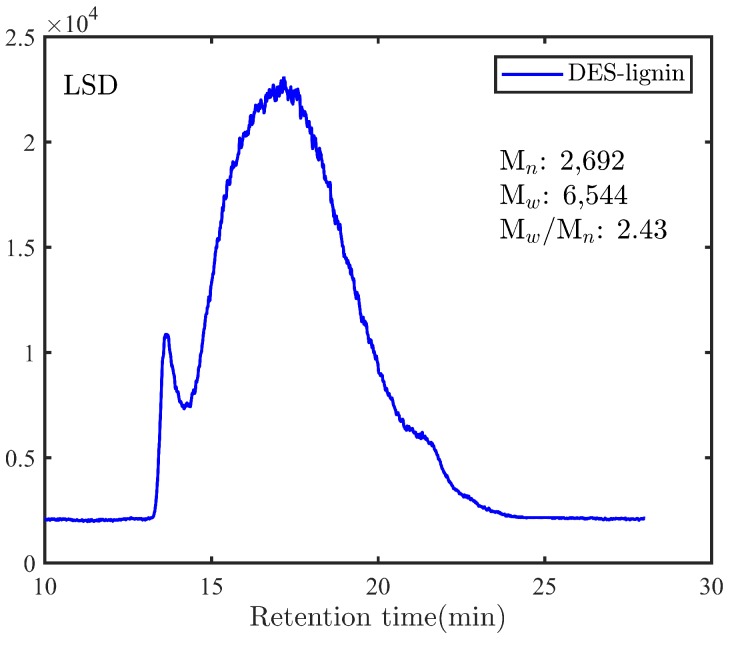
Molar mass distribution and average molecular weight of DES-lignin.

The molar mass distribution and the number average (M${}_{n}$) molecular mass of the extracted DES-lignin is presented in Figure 1.

The molecular weight of the DES-lignin was determined by using size-exclusion chromatography. The DES-lignin exhibited a weight-average (M${}_{w}$) molecular weight of 6544 g mol${}^{-1}$ and a number-average (M${}_{n}$) molecular weight of 2692, as can be seen in Figure 1. Furthermore, the DES-lignin displayed slim polydispersity, with an M${}_{w}$/M${}_{n}$ of 2.43, demonstrating further that the lignin was close to what is commonly believed to be its natural molecular weight [41].

### 3.2. Characterization of the Membranes

#### 3.2.1. FT-IR

**Figure 2:**
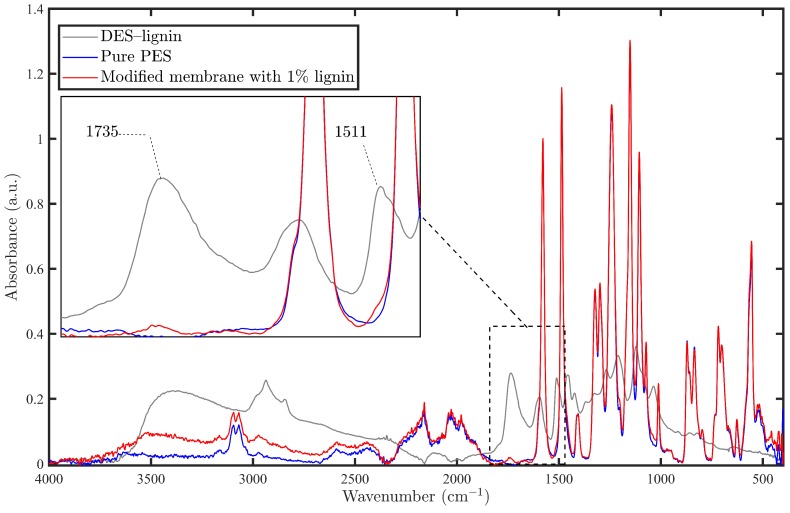
ATR-FTIR spectra of virgin and modified membrane with 1% DES-lignin in the region of 4000–400 cm${}^{-1}$. Non-overlapping peaks related to DES-lignin can be detected at 1511 cm${}^{-1}$, 1735 cm${}^{-1}$ and broad band between 3200 and 3600 cm${}^{-1}$.

The FT-IR spectra obtained from the original DES-lignin powder and the membrane modified with 1 wt % DES-lignin are compared with that of a pure PES membrane in Figure 2. Due to the low amount of DES-lignin powder (0.25 and 0.5 wt %) used in the modification of the membranes and overlapping of most of the DES-lignin peaks with the membrane peaks, the characteristic peaks of DES-lignin, the vibration of the unsaturated carbonyl group (C=O) at 1735 cm${}^{-1}$, and aromatic skeleton stretching (C=C) at 1511 cm${}^{-1}$ could not be unequivocally observed in the spectrum of the modified membranes. However, the new absorbance peaks could be seen clearly in the spectrum of the membrane prepared at the DES-lignin concentration of 1 wt %. At least two possible interactions, Π–Π interaction between benzene rings and hydrogen bonding between the hydroxyl group in DES-lignin and oxygen atoms of sulfone group in polyethersulfone, could be envisaged.

The presence of a strong and broad band between 3200 and 3600 cm${}^{-1}$ centering around 3500 cm${}^{-1}$, a slight peak at 1735 cm${}^{-1}$, and a shoulder peak at 1511 cm${}^{-1}$, which are associated to stretching vibrations of aromatic hydroxyl groups, carbonyl group, and coupled skeletal vibrations of the aromatic rings of DES-lignin, respectively, indicated the incorporation of DES-lignin within the polyethersulfone matrix. In addition, the area of C–H aromatic stretching vibration (3100–3000 cm${}^{-1}$) and aliphatic stretching vibration (2922–2875 cm${}^{-1}$) become more intense due the presence of DES-lignin in the structure of the modified membrane. The same behavior of interaction between a PES membrane and vanillin, as one of lignin derivatives, was also observed in our previous work [42] and in the study done by Virtanen et al., where the interactions between phenolic model compound vanillin and PES surface was comprehensively investigated [43]. They also mention that hydrogen bond formation between the hydroxyl group of this phenolic compound and ether oxygen of PES could also be established, although the hydrogen bonding with sulfur is the dominant interaction. As can be seen in Figure 2, the intensity of the ether group at 1043 cm${}^{-1}$ in the DES-lignin modified membrane becomes more intense compared to the virgin membrane, which is in accordance with the findings of Virtanen et al.

#### 3.2.2. Identification of Lignin by Color Measurement

**Figure 3:**
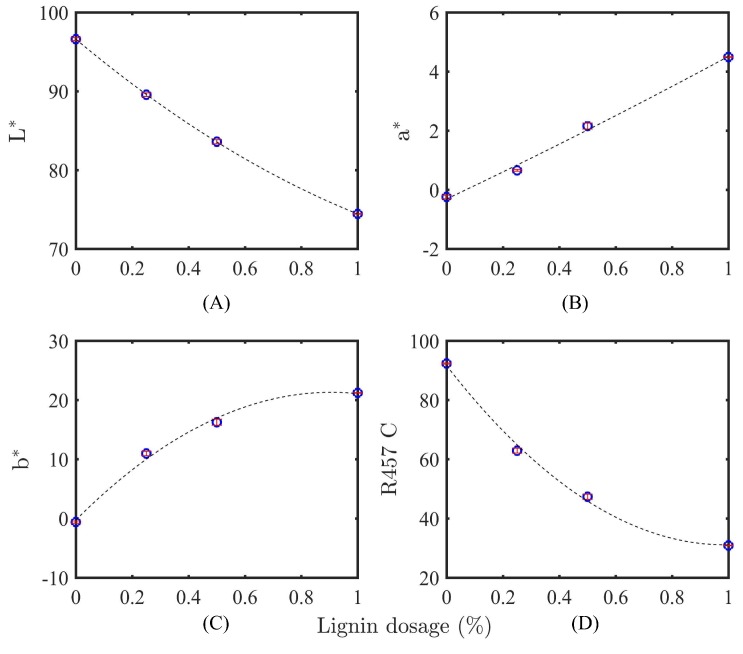
The effect of different lignin dosages (wt %) on the optical properties of fabricated membranes; (**A**) L${}^{*}$ (lightness/darkness), (**B**) a${}^{*}$ (greenness/redness), (**C**) b${}^{*}$ (blueness/yellowness), (**D**) ISO brightness (R457 C).

The L${}^{*}$ (lightness/darkness), a${}^{*}$ (greenness/redness), b${}^{*}$ (blueness/yellowness), and ISO brightness (R457 C) values of the fabricated membranes were measured and used as a tool to evaluate the lignin content used in membrane fabrication. As can be seen in Figure 3, the virgin membrane (without lignin) expresses the highest brightness (highest L${}^{*}$ and R457 C) and the lowest degree of blueness (b${}^{*}$). With a successive increase of lignin dosage in the dope solution, the brightness and b${}^{*}$ values of fabricated membranes reduced and increased remarkably, respectively. The reduction of brightness (the degree of yellowing) as a result of lignin and lignocellulosic compounds is mostly associated with the absorption of UV light by lignin chromophoric functional groups such as phenolics, hydroxyl, and carbonyl groups and double bonds that interact with UV light [44].

The results reflect the fact that the induction of lignin in the polymer solution leads to the formation of a darker membrane. These results are in agreement with Cheng et al. [45] and Du [46], who report the formation of a darker edible film as a result of lignin and phenolic compounds.

#### 3.2.3. Surface Charge

**Figure 4:**
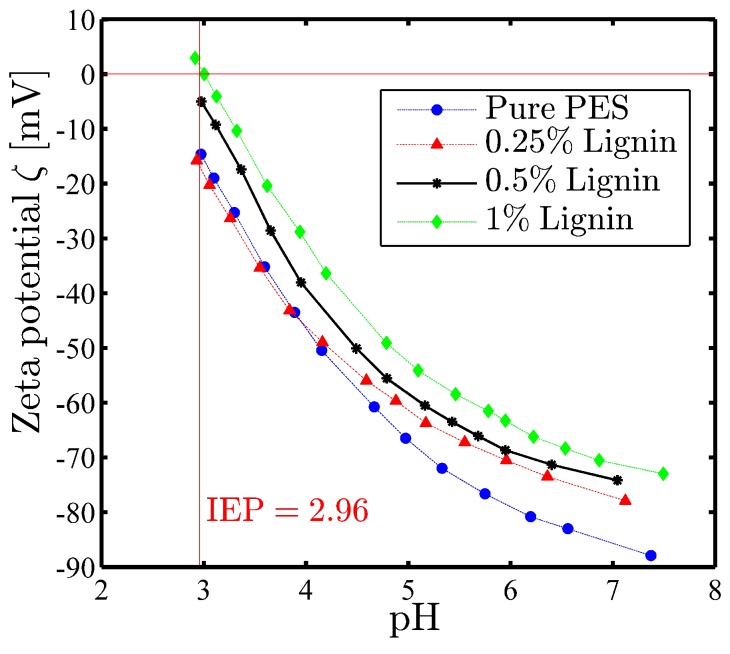
The effect of different wt % of lignin as an additive on the surface charge of modified membranes.

The zeta potential versus the pH of the reference and lignin-modified membranes are presented in Figure 4. As can be seen, the fabricated membranes possess generally a negatively charged surface over a wide pH range (3–8), which increases with a successive rise of pH. It can be seen clearly that membranes with less negative charge are attained as the dosage of lignin in the dope solution increases. The reduction of surface charge in the modified membrane compared to the virgin membrane can be explained by the fact that there are negatively and positively charged functional groups in the structure of lignin (phenolic, carboxyl, and methyl groups). Due to the interaction between lignin and the polyethersulfone polymer chain through hydrogen bonding, the available negatively charged group on the surface of the membrane can be reduced, and the zeta potential will thus decrease. In addition, it has been demonstrated in several studies that the lower absolute values of the zeta potential can stem from improvement in surface hydrophilicity [47,48], which is also in accordance with the contact angle (i.e., hydrophilicity) of fabricated membranes. The negative surface charge of lignin can be induced significantly by increasing the pH through different functional groups present in the structure of lignin [49,50]. Therefore, as can be seen in Figure 4, the surface charge of the modified membrane increases as the pH increases.

#### 3.2.4. SEM

**Figure 5:**
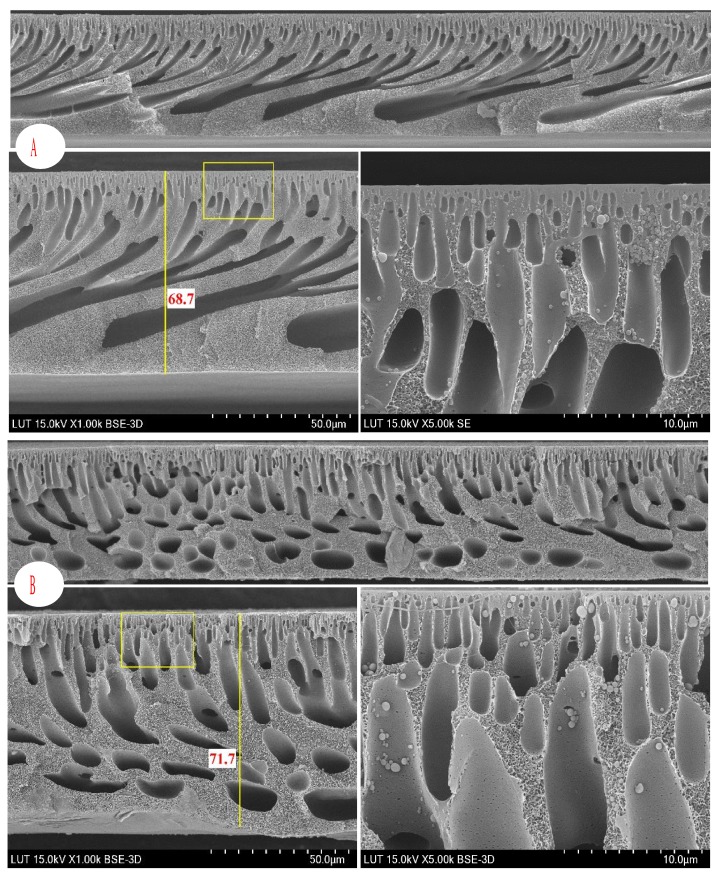

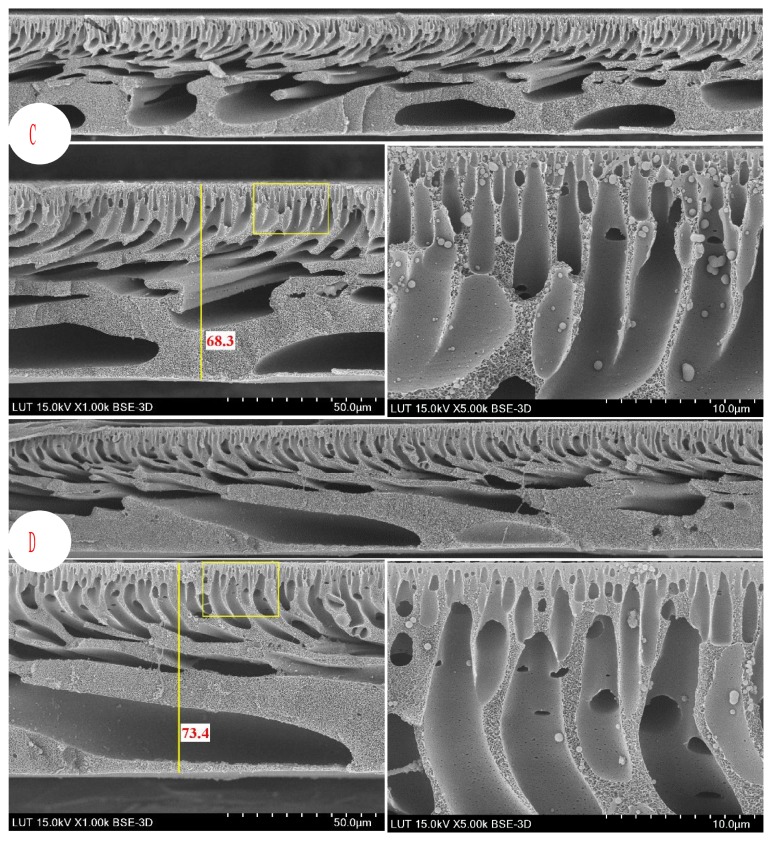
SEM cross-sectional images showing the effect of different amounts of added DES-lignin; (**A**) 0 wt %, (**B**) 0.25 wt %, (**C**) 0.5 wt %, (**D**) 1 wt %. The cross sections are on the left and the zoom sections of marked top layers are shown on the right.

Figure 5 shows the effect of various concentrations of DES-lignin (0–1 wt %) on the cross-section of the resultant membranes. The cross-section SEM images are represented in three magnifications (100, 50, and 10 μm) for better visualization. In general, all the fabricated membranes possess an asymmetric structure including a thin top skin layer, a porous middle sub-layer, and a bottom sub-layer. As can be seen in the SEM images, the sub-layers appear to possess cavities and morphologically different finger-like macrovoids in their structures. Kinetic hindrance and thermodynamic enhancement are of two most prominent factors which influence the ultimate morphology of membrane substantially, which in turn alters the membrane performance. In the case of the overall viscosity of the dope solution, no significant differences were found between the pure membrane and the modified ones, and it can thus be postulated that the kinetic effect of viscosity compared to the thermodynamic enhancement effect is minor. In general, the addition of non-solvent polymer additives in the initial casting solution reduces the thermodynamic miscibility of the casting solution with a non-solvent additive (i.e., increase of thermodynamic instability of the dope solution) and thus accelerates the diffusional exchange rate between the solvent (NMP:DMF) and the non-solvent (water). Furthermore, the presence of DES-lignin on the surface of the membrane increases the hydrophilicity of the membrane, which in turn enhances the diffusion of water as non-solvent into the cast film, and thereby a more open sub-layer can be formed in the membrane structure. As can be seen in the figures, the size and length of finger-like microvoids and cavities increases as the DES-lignin dosage increases in the dope solution. However, although the surface porosity and pore size distribution play determining roles in the membrane performance, the bulk porosity can also change the membrane permeability. ImageJ software (National Institutes of Health, Bethesda, MD, USA) was also used as a tool to estimate the bulk porosity of the membrane structure (cross-section) [51]. As can be seen in Table 1, the results of image analysis corroborate the increase of bulk porosity and the flux of fabricated membranes as a result of successive increase of DES-lignin in the dope solution. An increase of the bulk porosity of the membrane sub-layer as a result of incorporated alkali lignin into a polysulfone membrane has also been reported [16]. The presence of some trapped particles (0.2–0.5 μm) in the membrane matrix can be clearly seen in Figure 5 (zoom section of the top layer). This might be attributed to the precipitation of highly localized concentrated PES as a consequence of crystallization. The same crystallization-related precipitation conducts have been observed by Yeow et al. [52] for PVDF membranes. In fact, at a lower temperature, gelation prompted by crystallization arises first in the membrane formation processes and thus the crystals can grow as a result of delayed demixing [52].

### 3.3. Effect of DES-Lignin on the Antioxidant Activity of Fabricated Membranes

Antioxidative activity expressed as DPPH and ABTS radical scavenging activity of pure and modified membranes is presented in Table 1. In both antioxidant assays, as can be seen in the data in Table 1, the radical-scavenging activity of the fabricated membranes increased significantly (*p* < 0.05) as the DES-lignin content in the casting solution increased. In the membrane containing 1 wt % DES-lignin, the DPPH and ABTS antioxidant scavenging activity increased about 2.6- and 1.1-fold (the difference between antioxidant scavenging activities of membrane #4 and membrane #1 divided by antioxidant scavenging activity of membrane #1), respectively, in relation to the pure membrane sample. The better antioxidant activity of the DES-lignin membranes can be attributed to the presence of phenolic OH in the molecular structure of the DES-lignin, which can act as a hydrogen donor antioxidant [38]. The pure membrane also shows antioxidant activity to some extent, which may be attributed to the presence of the sulfone (O=S=O) group in the structure of the polyethersulfone membrane [53], which can act as electron donor in order to neutralize the free radicals by pairing the DPPH odd electron by electron donation.

### 3.4. Effect of DES-Lignin on Membrane Performance

The pure water flux of the reference and DES-lignin modified membranes at different operating pressure as well as their related PEG solution rejection (35 kD) and contact angles are presented in Table 2. It can be seen that the pure water flux increases as the dosage of DES-lignin in the dope solution increases, which is in accordance with the improvement of hydrophilicity, i.e., the lower contact angle of the modified membranes, and the bulk porosity of the fabricated membranes (Table 1). The better hydrophilicity in the membranes incorporated with DES-lignin can be ascribed to the presence of different hydrophilic functional groups in the DES-lignin structure, such as hydroxyl and carbonyl groups, as evidenced by a higher amplitude at wavenumbers of 3200–3600 and 1735 cm${}^{-1}$, respectively (Figure 2). These results are in line with the findings of Vilakati et al., which show a slight reduction of contact angles as a result of using alkali lignin as an additive in the modification of polysulfone membranes. Furthermore, the TOC results of pure water after 10 min (leakage) for each experiment was very low (below 1 mg/L), indicating no leakage of DES-lignin from the membrane structure. This trace of leakage can be attributed to the residual solvents remaining in the membrane structure, which could be seen in the reference membrane as well. In general, the inherent trade-off between permeability and rejection was obviated, and the PEG rejection for modified membranes remained almost the same as that of the reference membrane. Interestingly, in the low dosage of lignin content in the dope solution (0.25 wt %), a membrane with better performance in terms of pure water flux and PEG solution rejection was achieved.

## 4. Conclusions

DES-lignin was extracted successfully from birch wood by using a green solvent, a deep eutectic solvent, and characterized and incorporated into the polyethersulfone matrix to tailor the structure and performance of the membrane. The DES-lignin modified membranes revealed better hydrophilicity, antioxidant activity, and pure water permeability with a less negative surface charge compared to the pure membrane, while rejection stayed almost constant.

## Figures and Tables

**Table 1:** The effect of DES-lignin on the bulk porosity, DPPH and ABTS antioxidant activity of fabricated membranes.

Membrane	PES (%)	Lignin Content (%)	Bulk Porosity (%)	Antioxidant DPPH (%)	Capacities ABTS (%)
#1	20	-	69.3	19.3 ± 0.03	33.5 ± 0.1
#2	20	0.25	71.5	32.4 ± 0.06	42.5 ± 0.03
#3	20	0.5	72.5	43.1 ± 0.1	47.9 ± 0.06
#4	20	1	79.9	70.6 ± 0.2	71.7 ± 0.5

**Table 2:** Polymer solution condition, contact angle (±95% confidence Interval), pure water flux at different pressures, leakage (TOC results of pure water after 10 min), and PEG 35 kDa rejection of fabricated membranes.

Membrane	PES (%)	Lignin Content (%)	Contact Angle (${}^{{}^{\circ}}$)	Pure water Flux (L/m${}^{2}$h)			Permeability (L/m${}^{2}$h bar)	Leakage (mg/L)	Rejection (%) (PEG 35 kDa)
				1 bar	2 bar	3 bar			
#1	20	-	51.9 ± 0.4	67.7 ± 1.8	137.9 ± 0.7	210.0 ± 0.9	74.9	0.9	85.7
				75.9 ± 1.7	151.1 ± 0.5	233.3 ± 0.8		0.6	82.8
#2	20	0.25	48.6 ± 0.3	85.9 ± 2.0	171.2 ± 1.7	254.8 ± 1.4	81.5	0.5	88.7
				78.8 ± 1.9	154.5 ± 1.6	235.8 ± 0.9		0.4	84.9
#3	20	0.5	46.9 ± 0.4	94.2 ± 1.7	189.9 ± 0.7	286.0 ± 0.7	91.5	0.4	75.7
				86.4 ± 0.9	181.2 ± 0.8	260.8 ± 0.7		0.4	83.6
#4	20	1	45.6 ± 1.2	105.6 ± 1.3	206.0 ± 1.0	309.4 ± 0.9	96.9	0.6	86.6
				104.6 ± 1.4	183.7 ± 0.4	288.5 ± 0.5		0.8	81.4
